# Defective Suppressor of Cytokine Signaling 1 Signaling Contributes to the Pathogenesis of Systemic Lupus Erythematosus

**DOI:** 10.3389/fimmu.2017.01292

**Published:** 2017-10-16

**Authors:** Huixia Wang, Jiaxing Wang, Yumin Xia

**Affiliations:** ^1^Department of Dermatology, The Second Affiliated Hospital, School of Medicine, Xi’an Jiaotong University, Xi’an, China; ^2^Core Research Laboratory, The Second Affiliated Hospital, School of Medicine, Xi’an Jiaotong University, Xi’an, China

**Keywords:** suppressor of cytokine signaling 1, systemic lupus erythematosus, Janus kinase/signal transducer and activator of transcription pathway, cytokine, inflammation, lupus nephritis

## Abstract

Systemic lupus erythematosus (SLE) is a complex autoimmune disease involving injuries in multiple organs and systems. Exaggerated inflammatory responses are characterized as end-organ damage in patients with SLE. Although the explicit pathogenesis of SLE remains unclear, increasing evidence suggests that dysregulation of cytokine signals contributes to the progression of SLE through the Janus kinase/signal transducer and activator of transcription (STAT) signaling pathway. Activated STAT proteins translocate to the cell nucleus and induce transcription of target genes, which regulate downstream cytokine production and inflammatory cell infiltration. The suppressor of cytokine signaling 1 (SOCS1) is considered as a classical inhibitor of cytokine signaling. Recent studies have demonstrated that SOCS1 expression is decreased in patients with SLE and in murine lupus models, and this negatively correlates with the magnitude of inflammation. Dysregulation of SOCS1 signals participates in various pathological processes of SLE such as hematologic abnormalities and autoantibody generation. Lupus nephritis is one of the most serious complications of SLE, and it correlates with suppressed SOCS1 signals in renal tissues. Moreover, SOCS1 insufficiency affects the function of several other organs, including skin, central nervous system, liver, and lungs. Therefore, SOCS1 aberrancy contributes to the development of both systemic and local inflammation in SLE patients. In this review, we discuss recent studies regarding the roles of SOCS1 in the pathogenesis of SLE and its therapeutic implications.

## Introduction

As a complex autoimmune disease, systemic lupus erythematosus (SLE) is characterized by the presence of autoantibodies against self-antigens, including double-stranded (ds) DNA, as well as the risk of autoantibody-induced end-organ damage (1). Hematopoietic abnormalities, such as hyperactivation of T and B cells and overproduction of autoantibodies, exist in patients with SLE (2). Pathogenic autoantibodies may form immune complexes or directly deposit in the glomerular capillary, thus inciting irreversible glomerulonephritis, which is one of the most common complications in patients with SLE (3). In reality, numerous factors are implicated in the onset or progression of SLE. It is generally accepted that the deficiency in the clearance of apoptotic cells significantly contributes to the exposure of self-antigens, as well as subsequent autoimmune processes, such as autoantibody production and inflammatory responses (4). Albeit the precise mechanisms underlying SLE are yet to be elucidated, emerging evidences indicate that the abnormal expression of proinflammatory cytokines plays an important role in local inflammation and in the development of end-organ injuries in SLE (5).

Cytokines are central in both innate and adaptive immunity. They are mostly synthesized by immune cells and in turn participate in the differentiation, maturation, and activation of diverse immune and hematopoietic cells. Abnormalities of various cytokines have been identified in patients with SLE and in murine lupus models (5). Moreover, it was found that in sera of SLE patients, the level of transforming growth factor (TGF)-β is decreased; whereas certain proinflammatory cytokines, such as interferon (IFN)-α, IFN-γ, interleukin (IL)-6, IL-12, IL-17, IL-23, and B-cell activating factor (BAFF), are all upregulated accordingly (6). The dysregulation of these cytokines mirrors the imbalance in diverse immune cell subsets, such as T helper (Th) 1, Th2, Th17, and T regulatory (Treg) cells. Many cytokines can activate the Janus kinase 2 (JAK2)/signal transducer and activator of transcription 1 (STAT1) signaling pathway. Upon ligand binding, the activated JAK2 phosphorylates the cytoplasmic domains of cognate receptors, thus providing docking sites for STAT1 (7). Furthermore, STAT1 can also phosphorylate at the tyrosine site and form dimers before translocating into the nucleus, where the dimers activate target genes that are related to the development, differentiation, and survival of hematopoietic cells (8). The suppressor of cytokine signaling 1 (SOCS1) is an inhibitive factor induced by relevant cytokines, and it negatively regulates immune responses by suppressing the activity of JAK2 (8). Under normal conditions, the expression level of SOCS1 is minimal, but it can be rapidly upregulated in a feedback manner through the activation of JAK2/STAT1 signals (9, 10). As cytokine signaling ceases, SOCS1 is rapidly degraded (9). Therefore, SOCS1 is essential for maintaining immune homeostasis in local tissues.

Recent studies have demonstrated that SOCS1 participates in the pathogenesis of SLE (11, 12). The mRNA expression level of SOCS1 is significantly decreased in peripheral blood mononuclear cells of patients with SLE (11). Patients with active SLE have lower expression of SOCS1 mRNA as compared to patients with inactive SLE, hence indicating that mRNA expression of SOCS1 is negatively correlated with lupus disease activity (11). SOCS1 is also involved in other pathological processes of SLE including activation of immune cells, production of proinflammatory factors, initiation of renal fibrosis, etc. (12). The upregulation of SOCS1 through alternative methods is beneficial to improve the conditions of SLE patients (12, 13). In this review, we summarized recent studies on the function of SOCS1 in the pathogenesis of SLE and elaborated its clinical significance and therapeutic implications.

## The Structural Basis of SOCS1/JAK2 Interaction

**Figure 1:**
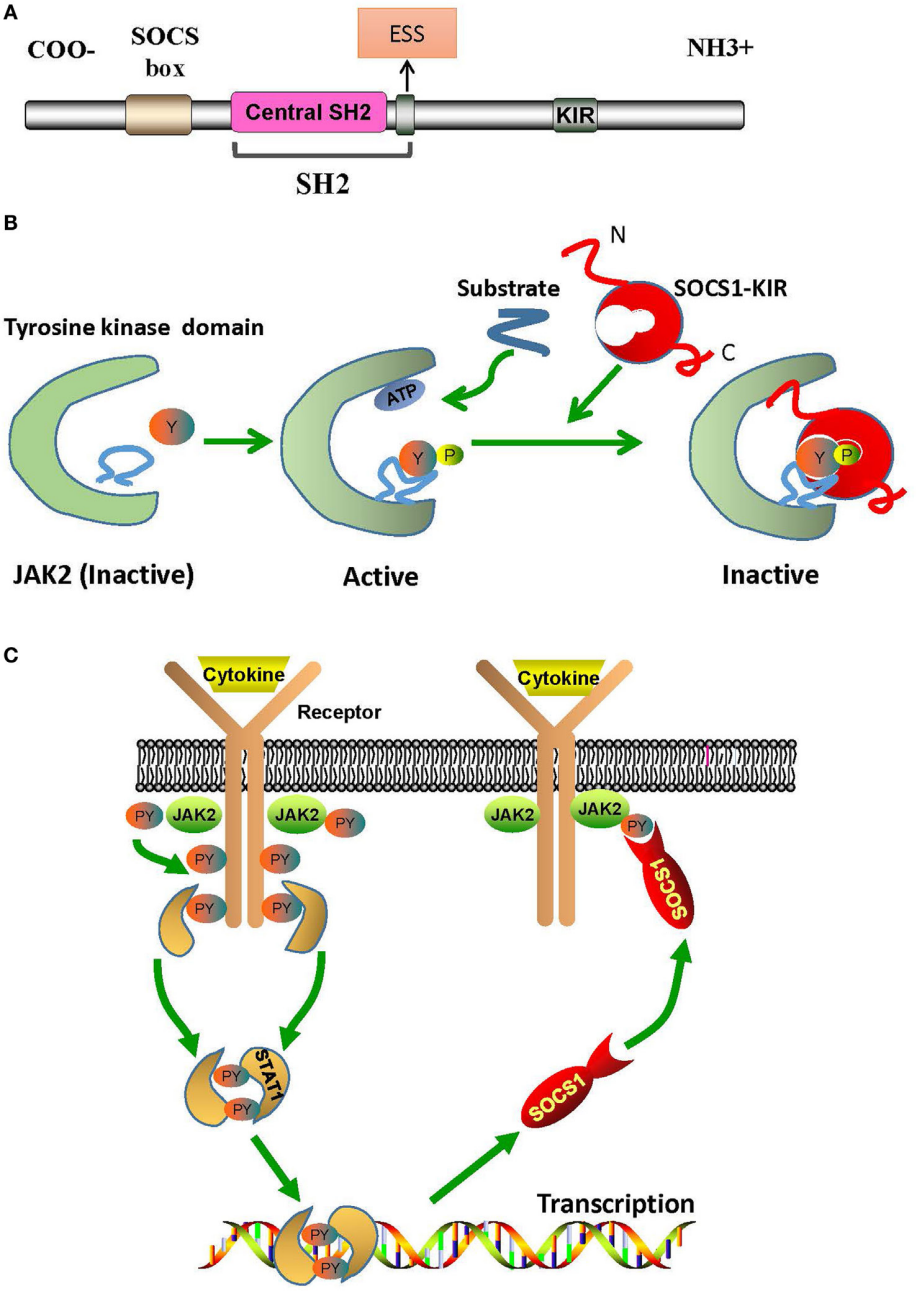
The structural basis of SOCS1/JAK2 interaction. **(A)** The SOCS1 protein contains a C-terminal SOCS box motif, SH2 domain, KIR region, and N-terminal region of varied length and amino acid composition. **(B)** Model of tyrosine kinase domain activation and inhibition by SOCS1. Binding of SOCS1-KIR to the activation loop prevents the access of substrates to the catalytic pocket. **(C)** Mechanism of negative cytokine signaling regulation by SOCS1 protein. Cytokine binding with specific cytokine receptors leads to receptor dimerization and subsequent recruitment of JAK2. Activated JAK2 phosphorylate the cognate cytokine-receptor cytoplasmic domain, providing docking binding sites for STAT1 proteins. After phosphorylation by JAK2, STAT1 proteins form dimers and translocate to the nucleus. STAT1 signaling induces SOCS1 protein transcription. Following their translation, SOCS1 proteins suppress cytokine signaling by binding to phosphorylated JAK2. ESS, extended SH2 subdomain; JAK2, Janus kinase 2; KIR, kinase inhibitory region; PY, phosphorylated tyrosine; SH2, Src-homology-region 2; SOCS1, suppressor of cytokine signaling 1; STAT1, signal transducer and activator of transcription 1.

The regulation of downstream signals by SOCS1 is triggered by the interaction between SOCS1-kinase inhibitory region (KIR) and JAK2 activation loop. Structurally, SOCS1 has an Src-homology-region 2 (SH2) with a central SH2 domain, an extended SH2 subdomain (ESS), and a KIR domain of 12 amino acids (Figure 1A) (10). The central SH2 domain has the most conserved sequence of SOCS1 protein. Mutations in the phosphotyrosine-binding residue Arg105 to Lys (R105K) or Glu (R105E) and deletion of the central SH2 domain can induce loss of function of SOCS1 (14). The ESS, comprising 12 residues, is essential for the interaction of SOCS1 with JAK2 (14). Substitution of conserved Ile68 with Glu (I68E) or Leu75 with Glu (L75E) in the ESS completely abolishes the binding of SOCS1 to JAK2 (14). KIR is responsible for the high-affinity binding of SOCS1 to the tyrosine kinase domain of JAK2, which further activates the kinase and transduces signals (10).

Janus kinase 2 is constitutively associated with the proline-rich, membrane proximal regions of cognate cytokine receptors, which are ubiquitously expressed in mammalian cells (15). Structurally, JAK2 can be roughly divided into an amino-terminal region (N region), followed by a catalytically inactive kinase-like domain and a tyrosine kinase domain (15). The N region participates only in cytokine receptor recognition and association; and the kinase-like domain of JAK2 is actually a pseudokinase domain that regulates only the catalytic activity of the tyrosine kinase domain, which is critical for the interaction of JAK2 with SOCS1 and for the catalytic activity of JAK2 as well (Figure 1B) (15). In humans, the amino acid sequence of autophosphorylation or activation loop in tyrosine kinase domain is ^1001^LPQDKEYYKVKEP, which is so called as pJAK2 (1001–1013) (16). pJAK2 has phosphorylated tyrosine 1007, which reflects the activation status of JAK2; and phosphorylation of this tyrosine leads to conformational changes that facilitate substrate binding (16). Moreover, structure–function studies have demonstrated that SOCS1 solely binds to phosphopeptides with phosphorylated tyrosine 1007 (14).

Kinase inhibitory region sequence is somehow similar to JAK2 activation loop (8). Thus, KIR may act as a pseudosubstrate and mimic the activation region of JAK2 to prevent substrate binding at the catalytic cleft of JAK2 (14). Substitution mutations in KIR, such as phenylalanine at positions 56 or 59, aspartic acid at 64, or tyrosine at 65, can reduce the ability of SOCS1 to bind with JAK2 activation loop (16). Hence, KIR combines with the JAK2 activation loop and induces conformational changes of the JAK2 activation site, thus abrogating the phosphorylation of substrates (17). After binding to phosphorylated JAK2, SOCS1 dephosphorylates and forms a complex, with JAK2, which then leads to irreversible JAK2 degradation (Figure 1C) (18). Under these interactions, the phosphorylation of STAT1 is hindered and immune responses, such as IFN-γ signaling, inflammatory factor production, T cell development and activation, etc., are then, therefore, suppressed (19, 20).

## The Principles of SOCS1 Regulation of Downstream Signals *In Vivo*

Insufficiency of SOCS1 expression and abnormalities in cytokine production are prominent in patients with SLE and in murine lupus models (6). A few *in vivo* studies have been carried out to reveal the effect of SOCS1 on downstream signals. IFN-γ plays an important role in patients with SLE, as it enhances the production of pathogenic autoantibodies and accelerates the progression of glomerulonephritis (21, 22). In fact, SOCS1 can directly bind to the IFN-γ receptor (IFNGR) to efficiently ensure the suppressive effect of SOCS1 on IFN-γ signaling, even at low levels of SOCS1 expression (23). Full inhibition of IFN-γ signaling by SOCS1 requires the phosphorylation of tyrosine 441 in the IFNGR1 subunit, thus suggesting that SOCS1 interacts first with the IFNGR and then binds to JAK2 to inhibit its kinase activity (24). Excessive production of IFN-γ and aberrant control of the IFN-γ signaling pathway have been implicated in the pathogenesis of SLE in BWF1 mice (25). In lupus-prone (NZB × NZW) F1 mice, SOCS1 expression was decreased, whereas pSTAT1 was increased in spleen-derived lymphocytes, thus mirroring the results of SOCS1 expression in peripheral blood mononuclear cells of patients with SLE (12). hCDR1 is a tolerogenic peptide derived from the sequence of the first complementarity-determining region (CDR1) of anti-DNA immunoglobulin (Ig) G, and it can downregulate pathogenic cytokines, such as tumor necrosis factor (TNF)-α, IL-1β, and IFN-γ, and upregulate the immunosuppressive cytokine TGF-β in lupus-prone mice (25). In these murine models, SOCS1 was upregulated upon subcutaneous administration of hCDR1, accompanied by pSTAT1 downregulation and tempered IFN-γ signaling (25). Moreover, patients undergoing prednisone treatment exhibited higher SOCS1 protein levels than those not receiving prednisone (12). Therefore, these findings suggest that SOCS1 insufficiency results in unbridled downstream signaling and contributes to the development and progression of SLE, whereas upregulation of SOCS1 definitely alleviates the course of SLE.

Extensive studies have revealed the crucial roles of IFN-α in the pathogenesis of SLE (26). Exposure to IFN-α *in vivo* can induce lupus disease in lupus-prone NZB/NZW F1 mice but not in BALB/c mice (27). Moreover, lupus-prone NZB mice lacking type I IFN receptor exhibited significant decrease in both autoimmunity and mortality (28). In SLE patients, serum IFN-α induce monocytes to differentiate into IFN-dendritic cells (DCs), which then capture apoptotic cells or nucleosomes and present these autoantigens to CD4^+^ T cells, thus initiating the proliferation of autoreactive T cells as well as the differentiation of autoantibody-producing B cells (29, 30). The dysregulation of IFN-α in SLE is also evident in gene expression profiles, including IFN-inducible genes, which correlate with the production of autoantibodies and the pathophysiology of SLE (31, 32). SOCS1 is an important inhibitor of IFN-α signaling *in vivo*. It associates with and regulates type I IFN receptor 1-specific signals, abrogates tyrosine phosphorylation of STAT1, and reduces the duration of antiviral gene expression—thus, SOCS1 balances the beneficial antiviral and detrimental proinflammatory effects of IFN-α (33). Furthermore, Toll/IL-1R-domain-containing adaptor protein inducing IFN-β–IFN-regulatory factor 3 pathway can rapidly induce type I IFN, which in turn activates secondary JAK/STAT1 pathway after stimulation with lipopolysaccharide and contributes, in combination with NF-κB, to the expression of IFN-inducible genes (34). Coincidentally, SOCS1 can effectively inhibit such process (35). Hence, SOCS1 deficiency in SLE patients might explain the excessive IFN-α signaling and high level of IFN-inducible genes, which subsequently contributes to the development of SLE.

Suppressor of cytokine signaling 1 deficiency is associated with the early death of mice, which were found to have severe lymphopenia, hyperactivation of peripheral T cells, fatty degeneration and necrosis of the liver, and inflammatory infiltration of liver and lungs (10). Partial restoration of SOCS1 can rescue Eμ-SOCS1^–/–^ mice from early onset of fatal diseases (36). However, these Eμ-SOCS1^–/–^ mice expressing insufficient SOCS1 spontaneously exhibited hyperactivation of T and B cells and DCs, produced anti-dsDNA antibodies, formed immune complexes in glomeruli, and eventually developed lupus-like disease (36). Moreover, SOCS1 deficiency induced prominent activation of STAT1, as well as hyperresponsiveness to IFN-γ, in mice models (12). However, IFN-γ deficiency can reverse the lupus phenotype of Eμ-SOCS1^−/−^ mice, which again suggests the negative regulation of IFN-γ signaling by SOCS1 (37).

**Figure 2:**
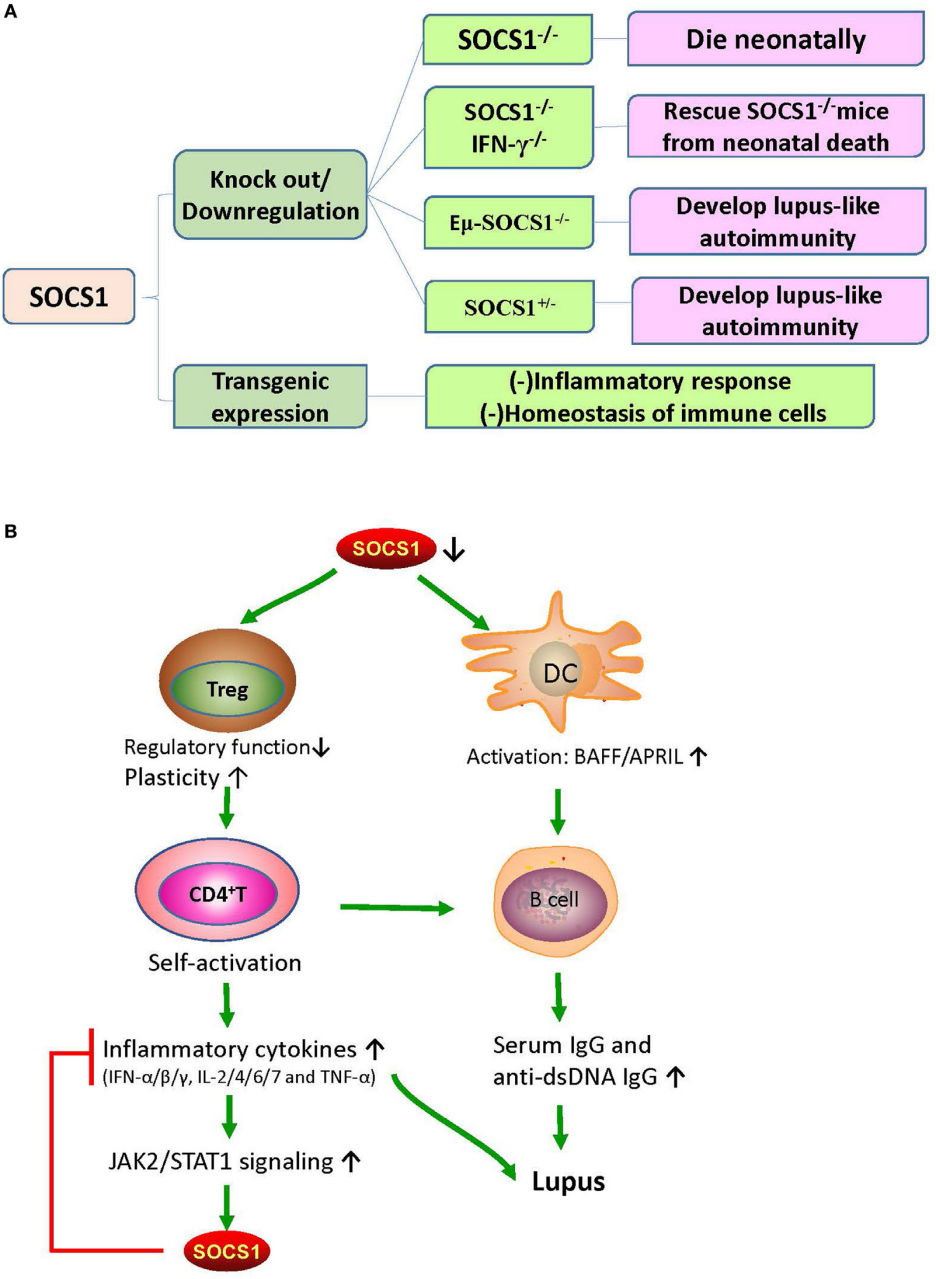
The effects of SOCS1 abnormalities on murine phenotypes and immune responses. **(A)** The effects of different SOCS1 level on the immune system of mice model. SOCS1^−/−^ mice died within 3 weeks after birth; and SOCS1^−/−^IFN-γ^−/−^ prevented the neonatal death of SOCS1^−/−^ mice, thus suggesting that uncontrolled IFN-γ signaling has destructive effects. Eμ-SOCS1^−/−^ and SOCS1^+/−^ mice developed lupus-like autoimmunity with age, indicating that SOCS1 deficiency can initiate an autoimmune response. However, transgenic overexpression of SOCS1 suppresses the immune response and disturbs the homeostasis of immune cells. **(B)** Roles of SOCS1 in systemic lupus erythematosus (SLE). There is a deficiency in the expression of SOCS1 in SLE. SOCS1-deficient DCs express high levels of BAFF, which leads to abnormal B-cell growth and proliferation. Moreover, low SOCS1 levels correlate with reduced suppressive capacity and enhanced plasticity of Treg cells. These Treg cells maintain high numbers of hyperactivated B cells by promoting the interaction of self-reactive CD4^+^ T cells with B cells. This interaction leads to the production of diverse inflammatory cytokines and autoantibodies, leading to immune complex formation and tissue injury. Therefore, upregulated SOCS1 levels might play a protective role through the suppression of the destructive response of inflammatory cytokines. APRIL, a proliferation-inducing ligand; BAFF, B-cell activating factor; DC, dendritic cell; IFN, interferon; IL, interleukin; JAK2, Janus kinase 2; SOCS1, suppressor of cytokine signaling 1; STAT1, signal transducer and activator of transcription 1; TNF-α, tumor necrosis factor alpha; Treg, T regulatory cells.

The SOCS1 transgenic mice were constructed by applying the *lck* proximal promoter to drive transgenic expression only in the T cell lineage (38). In these mice, tyrosine phosphorylation of STAT1 that is responsive to cytokines, such as IFN-γ, IL-6, and IL-7, was significantly suppressed; and the number of thymocytes decreased due to the blockade of development in the triple-negative stage, which consequently led to an increase in the percentage of CD4^+^ T cells (38). Moreover, in these mice, peripheral T cells were spontaneously activated, and apoptosis was significantly increased (38). These phenomena strongly suggest that SOCS1 maintains the homeostasis of peripheral T cells by suppressing STAT1 activation. The effects of SOCS1 abnormalities on murine phenotypes and immune responses are illustrated in Figure 2.

## SOCS1 Participates in the Hematologic Abnormalities in SLE

**Figure 3:**
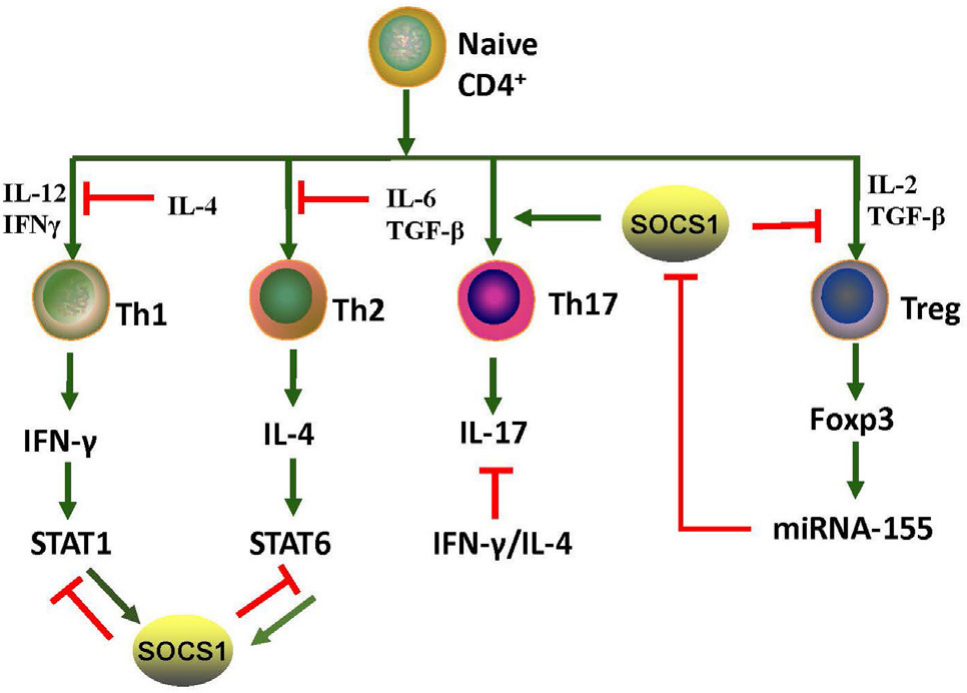
Roles of SOCS1 protein in CD4^+^ T cell differentiation. T cell differentiation from naïve cells into the various functional subtypes, namely Th1, Th2, Th17, and Treg cells, primarily depends on the effect of cytokines. SOCS1 inhibits the differentiation of Th1, Th2, and Treg but promotes Th17 differentiation. IL-4 secreted by Th2 induces SOCS1 expression of STAT6; and correspondingly, SOCS1 suppresses IFN-γ-STAT1 signaling of Th1. IFN-γ secreted by Th1 induces SOCS1 expression of STAT1; SOCS1, in turn, suppresses IL-4-STAT6 signaling of Th2. Therefore, SOCS1 positively regulates Th17 cell differentiation by inhibiting the antagonistic effects of IFN-γ and IL-4. SOCS1 is a target of miRNA-155, and its deletion would impair the function of Treg cells. Upregulation of Foxp3 promotes expression of miRNA-155, which accelerates the proliferative potential of Treg cells through SOCS1 downregulation. The red and green lines denote inhibitory and activating signaling, respectively. IFN, interferon; JAK2, Janus kinase 2; IL, interleukin; miRNA, microRNA; SOCS1, suppressor of cytokine signaling 1, STAT, signal transducer and activator of transcription; TGF-β, transforming growth factor beta; Th, T helper cells; Treg, T regulatory cells.

Eμ-SOCS1^–/–^ mice express only a limited level of SOCS1 in their peripheral lymphocytes, thus allowing the excessive activation of STAT1, development of multiple organ inflammation, spontaneous activation of lymphocytes, production of autoantibodies such as anti-dsDNA IgG, and development of prominent glomerulonephritis, which are all reminiscent of murine lupus models (36). Therefore, appropriate SOCS1 expression is critical for the prevention of systemic autoimmune disease such as SLE. CD4^+^ T cells were spontaneously activated in Eμ-SOCS1^–/–^ mice and in diseased SOCS1^+/−^ mice, and the T cells of SOCS1^+/−^ mice proliferated more significantly in response to IL-2 (36). In T cell-specific SOCS1-conditional knockout mice, SOCS1-deficient CD4^+^ naïve T cells mostly differentiated into Th1 cells, and Th17 differentiation was strongly suppressed (39); these mice eventually developed a lupus-like autoimmune disease (40). Previous studies have corroborated that Th1 polarization is primarily driven by IL-12 and IFN-γ, while Th2 polarization is primarily driven by IL-4 (41). As SOCS1 suppresses both IFN-γ and IL-4 signaling, SOCS1 upregulation may be an approach for the reciprocal inhibition of Th1 and Th2 cells. When IFN-γ is excessively expressed, IL-4 signaling through STAT6 can be blocked by SOCS1; however, when IL-4 is highly expressed, IFN-γ signaling through STAT1 is blocked by SOCS1 (39). Both IL-6 and TGF-β promote the production of IL-17 by naïve CD4^+^ T cells, and this is essential for the development and differentiation of Th17 cells (42, 43). SOCS1^−/−^ T cells are less responsive to TGF-β, and this possibly explains the inhibition of Th17 differentiation of SOCS1^−/−^ T cells (39). Moreover, early differentiation of Th17 cells is inhibited by IFN-γ and IL-4 by depressing the production of IL-17 (39). Therefore, SOCS1 deficiency highly contributes to the imbalance of different Th cells and subsequent autoimmunity (Figure 3).

Suppressor of cytokine signaling 1 inhibition is important in the pathogenesis of SLE through the promotion of Treg cells plasticity (44). Dysregulation of Treg cells is highly implicated in the pathogenesis of SLE (45). T cell activation and autoantibody expression are accelerated in Treg cell-depleted lupus-prone mice (46). Transfer of Treg cells from normal mice into the murine lupus model can effectively suppress the progression of lupus autoimmunity such as anti-dsDNA antibody generation and lupus nephritis (LN) (47). However, SOCS1-deficient Treg cells usually lose Foxp3 expression and are converted into Th1-like cells, and this can be attributed to the hyperresponsiveness of Treg cells to IL-2 and IFN-γ, in which both accelerate the proliferation of Treg cells and its conversion into effector cells (48). As previously described, T cell-specific SOCS1-conditional knockout mice developed lupus-like diseases including spontaneous dermatitis, splenomegaly, lymphadenopathy, and serum positivity of anti-dsDNA antibodies (40). Treg-specific SOCS1-deficient mice also developed lupus-like phenotypes that are less serious than those in T cell-specific SOCS1-deficient mice, and many adult SOCS1^+/−^ mice exhibited lupus-like manifestations as well (36). Splenic Treg cells from diseased SOCS1^+/−^ mice showed less suppressive functions upon self-reactive T and B cells (36). Therefore, SOCS1 plays a crucial role in the interference of SLE development by maintaining the suppressive functions of Treg cells and by preventing Treg cells plasticity.

Suppressor of cytokine signaling 1 regulates the maturation and activation of DCs. In Eμ-SOCS1^–/–^ mice, DCs expressed higher levels of costimulatory molecules, such as CD80 and CD86 (36). Moreover, SOCS1-deficient DCs secreted more proinflammatory cytokines, such as IFN-γ, IL-6, IL-12, and TNF-α, and higher levels of major histocompatibility complex (MHC) class II molecules upon stimulation with lipopolysaccharide and CpG-containing DNA (49, 50). DCs are implicated in the development of systemic autoimmunity in aged SOCS1^−/−^ mice (19). Transfer of SOCS1^−/−^ DCs to wild-type mice induced the generation of autoantibodies due to the overexpression of BAFF in the donor DCs (19). It is known that failure of autoimmune tolerance accelerates the development of SLE (51). Self-tolerance can be disrupted by excessive IL-12 production of SOCS1^−/−^ DCs (50). Therefore, SOCS1 inhibition participates in the pathogenesis of SLE by favoring the activation of DCs.

Systemic lupus erythematosus is characterized by serum positivity of anti-dsDNA autoantibodies, which are produced by B cell-derived plasma cells (52). Anti-dsDNA autoantibodies are instrumental in LN through recognition of multiple self-antigens and initiation of renal fibrosis (53–55). BAFF, which is a DC- and monocyte-derived cytokine of TNF family, is crucial in regulating B cell maturation, survival, and function (56). The expression level of BAFF/BLyS (B-lymphocyte stimulator) is increased in MRL/lpr mice during the onset and progression of lupus-like diseases (57). BAFF/BLyS-transgenic mice also had elevated serum titers of Ig and developed lupus-like autoimmunity (58, 59). Interestingly, high levels of BAFF/BLyS were detected in DCs but not in macrophages of SOCS1^−/−^ transgenic mice, wherein transgenic SOCS1 was expressed in T and B cells but not in DCs (19). Furthermore, DCs induce Ig class switching through BLyS and a proliferation-inducing ligand (APRIL) (60). When BAFF/BLyS and APRIL are blocked by soluble B cell maturation antigen-Fc, as well as transmembrane activator and CAML interactor-Fc, the generation of IgG1 by B cells is partially restricted in the presence of SOCS1^−/−^ DCs (60). Lipopolysaccharide induced more anti-dsDNA antibodies in the sera of C57BL/6 mice after they received DCs from SOCS1^−/−^ transgenic mice (19). Thus, SOCS1 inhibition facilitates the activation of DCs, increases autoantibody generation and Ig class switching, and promotes the occurrence and development of SLE.

SOCS1 polymorphisms may also contribute to the development of SLE. It was found that the SLE patients have a lower frequency of SOCS1-1478del compared with those SLE patients without thrombocytopenia (61), suggesting that genetic background influences specific hematologic abnormalities in patients with SLE through regulating SOCS1 gene expression.

## SOCS1 Inhibition is Pivotal in the Pathogenesis of LN

Lupus nephritis is one of the most common complications in patients with SLE (3). It is primarily induced by renal deposition of pathogenic autoantibodies including anti-dsDNA IgG. It also involves the infiltration of immune cells, such as macrophages and lymphocytes, as well as the production of proinflammatory and profibrotic cytokines, namely IL-6, IL-12, IFN-γ, TNF-α, TGF-β, and monocyte chemoattractant protein-1, which accelerate renal injuries (21). In progressive LN, fibrosis is one of the main pathologies, and it contributes to the development of end-stage renal disease, which is evidenced by glomerular sclerosis (3). Wang et al. have demonstrated that SOCS1 expression is decreased in the glomeruli of LN patients and in MRL/lpr mice with anti-dsDNA IgG deposition as compared with their control groups (53). In MRL/lpr mice, STAT1 is overexpressed in glomerular mesangial, endothelial, and tubular epithelial cells, whereas SOCS1 is downregulated accordingly (62). In rat model of rapid focal segmental glomerulosclerosis, the expression levels of α-smooth muscle actin, collagen IV, and TGF-β1 were increased in the kidneys and were accompanied by reduced SOCS1 expression and activated JAK2/STAT1 signals (63).

Previous studies have shown that anti-dsDNA IgG binds to cell surface molecules, directly penetrates into kidney cells, and facilitates cell proliferation in the kidney (64). In addition, anti-dsDNA IgG participates in renal fibrosis through the induction of a myofibroblast-like phenotype of mesangial cells, as well as the production of proinflammatory cytokines and fibrotic factors in renal cells (65). Moreover, anti-dsDNA IgG can effectively catalyze DNA or peptides, depending on the structure of self-antigens (52). An interesting phenomenon is that anti-dsDNA IgG exhibits nephritogenicity through the blockade of SOCS1 signals. Anti-dsDNA IgG specifically binds to SOCS1-KIR and directly catalyzes KIR (53). Therefore, anti-dsDNA IgG competes with JAK2 activation loop for KIR, which leads to the blockade of signals from SOCS1 to the JAK2/STAT1 pathway. Downstream proinflammatory cytokines and profibrotic factors are upregulated in LN (53). In addition, ALW, which is a DNA-mimicking peptide with a sequence of ALWPPNLHAWVP, can restore SOCS1 expression by blocking the binding of anti-dsDNA IgG to antigens, thus further suppressing the JAK2/STAT1 pathway and attenuating LN (53, 54). These findings suggest that SOCS1 is involved in the nephritogenicity of anti-dsDNA IgG and that SOCS1 upregulation can ameliorate LN.

MicroRNAs (miRs) have been implicated in the pathogenesis of renal fibrosis (66). In patients with LN, miR-150 is overexpressed in resident cells of kidneys (67). SOCS1 is one of the potential targets of miR-150 (68). In proximal tubular and mesangial cells *in vitro*, miR-150 inhibited SOCS1 expression and increased the production of profibrotic proteins such as fibronectin, collagens I and III, and TGF-β1 (68). In podocytes, TGF-β stimulates miR-150 expression, accompanied by decreased SOCS1 and increased COL1 and COL3 expression (68). These findings are consistent with the facts that SOCS1 acts as an attenuator of renal immune responses, tubular epithelial–myofibroblast transdifferentiation, and tubulointerstitial fibrosis (69). Moreover, macrophage infiltration is a prominent feature of glomerulonephritis (70). SOCS1 regulates M1-macrophage activation, which mainly mediates inflammation and tissue damage by inhibiting IFN-γ-induced JAK2/STAT1 signaling in LN (71, 72). M1 macrophages of SOCS1-knockdown mice produced increased levels of proinflammatory cytokines, such as IL-6, IL-12, and MHC class II molecules, thus suggesting that SOCS1 limits the proinflammatory characteristics of M1 macrophages and regulates inflammatory balance (73).

**Figure 4:**
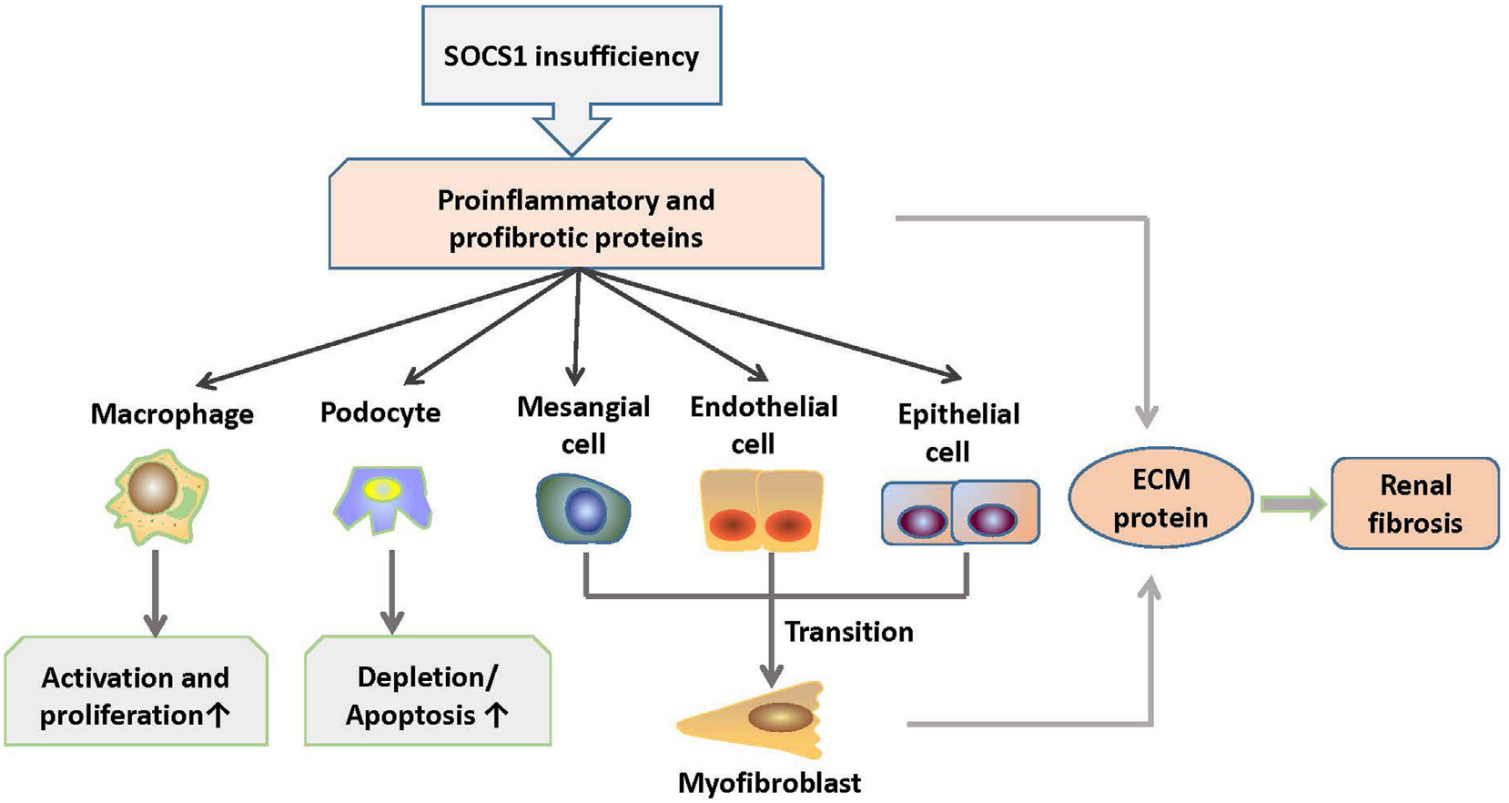
Roles of SOCS1 in the pathogenesis of LN. SOCS1 deficiency results in excessive production of proinflammatory and profibrotic molecules, which further induces increased activation and proliferation of macrophages (M1) and depletion and apoptosis of podocytes. In addition, molecules, such as TGF-β, cause mesenchymal transition of renal cells and overexpression of ECM proteins in the kidney, which contribute to renal fibrosis. ECM, extracellular matrix; SOCS1, suppressor of cytokine signaling 1.

Diabetic nephropathy is characterized by inflammation of the glomeruli and tubulointerstitial regions, accumulation of extracellular matrix (ECM), and subsequent focal and global glomerular sclerosis (74). Kidney infiltration of M1 macrophages in diabetic mellitus patients exacerbates renal cell damage (75). Numerous studies have demonstrated that dysregulated JAK/STAT signaling contributes to the onset and progression of diabetic chronic vascular complications, such as nephropathy (76). Interestingly, these features are similar to LN in a certain degree. Intraperitoneal administration of SOCS1 peptidomimetic (^53^DTHFRTFRSHSDYRRI^68^), which is a peptide that mimics the activity of the SOCS1 KIR region, in mice with diabetic nephropathy suppressed the activation of STAT1 signals, reduced serum creatinine and albuminuria levels, and ameliorated mesangial expansion, tubular injury, and renal fibrosis (77). Moreover, these SOCS1 peptidomimetic-treated mice exhibited significantly decreased T lymphocytes and M1 macrophages infiltration and reduced expression levels of monocyte- or T cell-derived chemokines such as C chemokine ligand (CCL) 2, CCL5, and TNF-α (77). Furthermore, SOCS1 peptidomimetic inhibits the expression of target genes induced under inflammation and reduces the migration and proliferation of mesangial and tubuloepithelial cells (77). Therefore, the correction of SOCS1 expression may be a promising method to suppress the development of inflammatory nephropathy such as LN. The role of SOCS1 signaling in the pathogenesis of LN is shown in Figure 4.

## SOCS1 Signals are Involved in Other End-Organ Injuries in SLE

**Figure 5:**
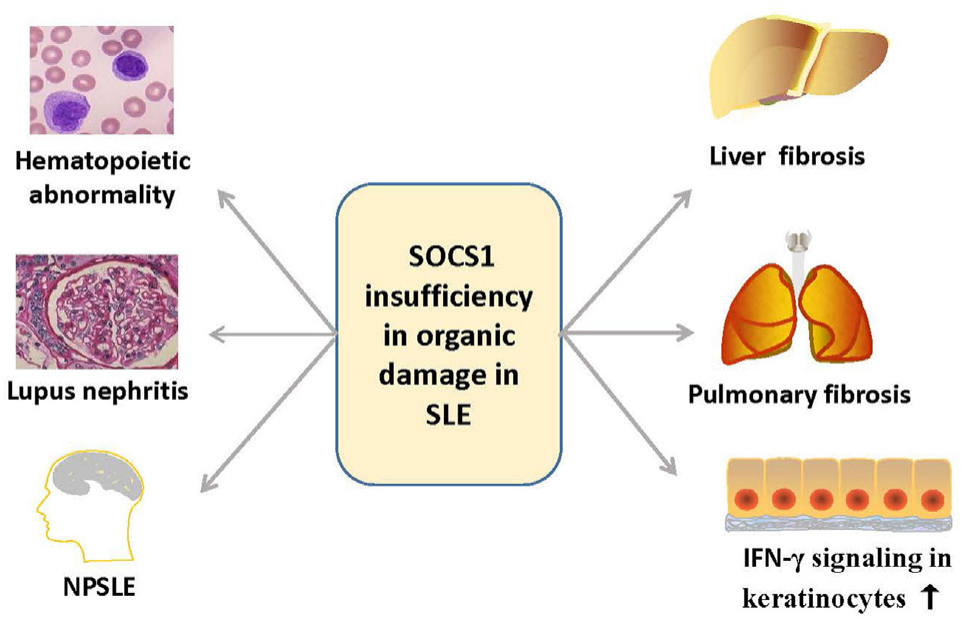
Overview of the organ damage in systemic lupus erythematosus related with SOCS1 insufficiency. SOCS1 insufficiency contributes to the hematopoietic abnormalities such as spontaneous lymphocyte activation, production of autoantibodies, lupus nephritis, NPSLE, and liver or pulmonary fibrosis, and it also promotes autoimmune responses in skin *via* IFN-γ signaling. IFN, interferon; NPSLE, neuropsychiatric systemic lupus erythematosus; SOCS1, suppressor of cytokine signaling 1.

Suppressor of cytokine signaling 1 is also involved in the function and injuries of other organs such as skin, central nervous system, liver, and lungs (78–80) (Figure 5). Cutaneous manifestations appear in most patients with lupus erythematosus, and IFN-γ is essential for the autoimmune responses in the skin of these SLE patients, as keratinocytes are highly susceptible to IFN-γ (81). Upon stimulation by IFN-γ, keratinocytes produce diverse chemokines, such as CCL2 and chemokine C–X–C motif ligand 10 (CXCL10), which promote the immigration of T cells, monocytes, and DCs into the inflamed skin, and CXCL8, which drives the chemoattractant activity of neutrophils (79, 81). However, SOCS1 suppresses the effect of IFN-γ on keratinocytes by inhibiting the JAK2/STAT1 pathway (79, 82). Keratinocytes overexpressing SOCS1 are less responsive to IFN-γ, as mirrored by the decreased activation of STAT1 and lowered production of CCL2, CXCL10, intercellular adhesion molecule-1, and MHC class II molecules (83). Evidently, SOCS1 protects keratinocytes of SLE patients from autoimmunity induced by uncontrolled IFN-γ signaling.

Neuropsychiatric SLE (NPSLE) is a serious complication of SLE (84). Although the mechanism of NPSLE remains unclear, cytokines and chemokines, such as IFN-β and IFN-γ, are considered to be involved in the pathogenesis of NPSLE through the JAK/STAT signaling pathway (85–88). IFN-β-treated astrocyte *in vitro* was able to generate a large amount of chemokines, such as CCL2, CCL5, and CXCL10, and these chemokines can be negatively regulated by SOCS1 (89). The production of these chemokines apparently increased when SOCS1 is depleted by siRNA (78). Furthermore, the increase in chemokine expression correlates with enhanced migration of macrophages and CD4^+^ T cells *in vitro*, indicating that SOCS1 might limit inflammatory cell migration within the central nervous system (89). Moreover, SOCS1 also inhibits IFN-γ-induced expression of MHC class II and CD40 in macrophages and microglia by blocking STAT1 activation (90, 91). Thus, SOCS1 inhibition contributes to the autoimmunity in the progression of NPSLE by affecting the production of inflammatory cytokines and chemokines, activation of microglia, macrophages and astrocytes, and infiltration of immune cells.

Aside from kidneys, skin, and central nervous system, the liver can also be affected in SLE (92). About 25–50% of SLE patients may present with abnormal liver function (93). Many studies demonstrated the ability of antiribosomal P antibodies to upregulate the expression of proinflammatory cytokines produced by peripheral monocytes in SLE, which can lead to the development of autoimmune hepatitis (94). In patients with hepatitis triggered by SLE, Treg cells are decreased in number and display impairment of suppressive function, along with elevated IFN-γ production *in vivo* (95). Diseased SOCS1^+/−^ mice exhibited more severe liver fibrosis than wild-type littermates. Liver fibrosis is strongly correlated with SOCS1 gene silencing through DNA methylation, and this firmly supports that the inhibition of SOCS1 leads to the progression of autoimmune hepatitis in SLE (96).

Furthermore, a variety of cytokines and chemokines are involved in the pathophysiology of pulmonary fibrosis in SLE (97, 98). It was reported that lower levels of SOCS1 mRNA and higher amounts of type I collagen were produced by fibroblasts from lungs of patients with pulmonary fibrosis as compared with those from healthy lungs (99). Moreover, SOCS1 deficiency in murine fibroblasts resulted in increased collagen production, whereas overexpression of SOCS1 suppressed collagen expression *in vitro* (99). Therefore, SOCS1 might suppress pulmonary fibrosis by inhibiting profibrotic cytokines and collagen synthesis of lung fibroblasts. The expression level of SOCS1 in bleomycin-injured lungs was significantly lower in SOCS1^+/−^ mice than in wild-type mice (100). SOCS1^+/−^ mice treated with bleomycin had significantly increased numbers of macrophages, lymphocytes, and eosinophils and elevated levels of IFN-γ, TNF-α, IL-4, IL-5, and monocyte chemoattractant protein-1 as compared with those of SOCS1^+/+^ mice in bronchoalveolar lavage fluid (100). Exogenous SOCS1 delivered through adenoviral gene transfer ameliorated bleomycin-induced pulmonary inflammation and fibrosis in SOCS1^+/−^ mice (100). These results highly suggest that SOCS1 inhibition is also involved in the progression of pulmonary fibrosis and that SOCS1 would be a novel target in treating lung fibrosis. The roles of SOCS1 in the different forms of lupus erythematosus are summarized in Table 1.

**Table 1:** The roles of SOCS1 deficiency in systemic lupus erythematosus (SLE).

Affected	Phenotype	Target	Effect	Reference
Hematological system	Hematopoietic abnormalities	Th cells	Spontaneous activation and proliferation; Th1↑/Th17 ↓	(36, 39)
		Treg cells	Cells plasticity ↑	(36, 44, 48)
		Dendritic cells	Activation; BAFF ↑	(19, 36, 49, 50)
		Autoantibody	IgG ↑; Ig class switching ↑	(19, 60)

Kidney	Lupus nephritis	Anti-dsDNA IgG	Binds and catalyzes SOCS1-KIR	(53)
		miRNA-150	SOCS1 expression ↓; renal fibrosis	(67)
		Macrophages	Renal inflammation	(70–72)

Skin	Cutaneous inflammation	Keratinocytes	Interferon-γ signaling ↑	(78, 81)

Brain	Neuropsychiatric SLE	Astrocytes	Activation ↑, inflammatory cytokines and chemokines ↑	(88–90)
		Microglia		
		Macrophages		
		T cells		

Liver	Lupus hepatitis			(95)

Lung	Pulmonary fibrosis	Macrophages	Activation ↑, profibrotic cytokines ↑, collagen synthesis ↑	(98, 99)
		Lymphocyte		

*BAFF, B-cell activating factor; KIR, kinase inhibitory region; SOCS1, suppressor of cytokine signaling 1; Th, T helper cells; Treg, T regulatory cells*.

## Therapeutic Potential for Targeting the SOCS1 Pathway

Considering the abnormalities of SOCS1 expression in damaged tissues, as well as its role in the regulation of downstream cytokines, SOCS1 may be a novel therapeutic target in the treatment of patients with SLE. Administration of SOCS1 mimetics might affect the abnormal immune responses regulated by SOCS1. Tyrosine kinase inhibitory peptide (Tkip), which is a 12-amino acid peptide (WLVFFVIFYFFR), can specifically bind to the JAK2 activation loop (^1001^LPQDKEYYKVKEP) and inhibit the activation of JAK2/STAT1 signaling (101). *In vivo* studies have demonstrated that subcutaneous administration of Tkip can block IFN-γ and TNF-α pathways and prevent the development of experimental autoimmune encephalomyelitis and multiple sclerosis (102). Moreover, the SOCS1-KIR mimetic peptide PS-5 (^53^DTHFRTFRSHSDYRRI) ameliorates IFN-γ-induced inflammation in human keratinocytes by suppressing JAK2 kinase activity, as reflected by the inhibition of STAT1α phosphorylation and reduced expression of IFNGR1, CCL2, CXCL10, and intercellular adhesion molecule-1 (79). These strategies suggest that administration of SOCS1 mimetics is capable of ameliorating SLE.

hCDR1 (Edratide), a peptide (GYYWSWIRQPPGKGEEWIG) based on the CDR1 sequence of anti-DNA monoclonal antibody, could ameliorate the progression of SLE (13). In SLE patients treated with Edratide subcutaneously, the expression of pathogenic cytokines, such as IL-1β, TNF-α, IFN-γ, and BLyS, were significantly downregulated, but the expression of anti-inflammatory cytokine TGF-β was increased (13). After the administration of hCDR1, NZB × NZW F1 mice showed increased SOCS1, decreased levels of pSTAT1, BAFF, anti-dsDNA autoantibodies, and MHC class II molecules on DCs, and better controlled IFN-γ signaling (25). Clinically, glomerular immune complex deposit was diminished and proteinuria levels were reduced in these lupus-affected mice upon injection of hCDR1 (25). It is, therefore, possible that part of the beneficial effects of hCDR1 is due to the induction of SOCS1 in hCDR1-treated mice and controlled IFN-γ signaling (25). The therapeutic strategies for targeting SOCS1 pathway are also summarized in Table 2.

**Table 2:** Therapeutic potential for targeting SOCS1 pathway.

Pattern	Approach	Function	Mechanism	Implications	Reference
Upregulation of SOCS1	Adenoviral gene transfer	SOCS1 delivery	Upregulating SOCS1	Bleomycin-induced pulmonary fibrosis in SOCS1^+/−^ mice	(99)
	Tyrosine kinase inhibitory peptide	SOCS1 mimetic	Competitive binding to the activation loop of JAK2	Experimental autoimmune encephalomyelitis and multiple sclerosis	(100, 101)
	PS-5	SOCS1-KIR analog	Competitive binding to the activation loop of JAK2	Psoriasis	(79)
	Edratide	SOCS1 inductor: a drug based on the CDR1 sequence of anti-DNA IgG		Systemic lupus erythematosus	(13, 25)

*CDR1, complementarity-determining region 1; JAK2, Janus kinase 2; KIR: kinase inhibitory region; SOCS1, suppressor of cytokine signaling 1*.

Thus far, we have yet to fully understand the function of SOCS1 *in vivo*, because intracellular signaling pathways are complexly regulated by various factors. With the development of new technologies, the roles of SOCS1 in SLE will be explicitly elucidated, and SOCS1 signals can provide more therapeutic strategies for the treatment of SLE in the future.

## Conclusion

The SOCS1 pathway is a key regulator of inflammatory cytokines, which are pivotal in the progression of SLE. The insufficient expression of SOCS1 in SLE is related with various pathological processes including hematologic abnormalities, generation of autoantibodies, and other end-organ damages such as LN. Although the explicit role of SOCS1 remains to be elucidated, SOCS1 insufficiency definitely contributes to the pathogenesis of SLE. The enhancement of SOCS1 signals, such as SOCS1 delivery or SOCS1 mimetics, can ameliorate the manifestations of SLE. Further investigation should focus on the design of SOCS1-mimicking molecules that may rectify SOCS1 insufficiency in SLE.

## Author Contributions

YX and HW conceived this review paper and prepared the manuscript. JW discussed the manuscript and contributed to the improvement of this paper. All the authors read and approved the final manuscript.

## Conflict of Interest Statement

The authors declare that the research was conducted in the absence of any commercial or financial relationships that could be construed as a potential conflict of interest.
